# Prediction of solid and micropapillary components in lung invasive adenocarcinoma: radiomics analysis from high-spatial-resolution CT data with 1024 matrix

**DOI:** 10.1007/s11604-024-01534-2

**Published:** 2024-02-28

**Authors:** Keisuke Ninomiya, Masahiro Yanagawa, Mitsuko Tsubamoto, Yukihisa Sato, Yuki Suzuki, Akinori Hata, Noriko Kikuchi, Yuriko Yoshida, Kazuki Yamagata, Shuhei Doi, Ryo Ogawa, Yukiko Tokuda, Shoji Kido, Noriyuki Tomiyama

**Affiliations:** 1 Department of Radiology, Osaka University Graduate School of Medicine, 2-2 Yamadaoka, Suita, Osaka 565-0871 Japan; 2 Nishinomiya Municipal Central Hospital, 8-24 Hayashidacho, Nishinomiya, Hyogo 663-8014 Japan; 3 Suita Municipal Hospital, 5-7 Kishibeshinmachi, Suita, Osaka 564-0018 Japan

**Keywords:** Lung adecocarcinoma, Micropapillary-component, Solid-component, Radiomics, Highspatial-resolution CT

## Abstract

**Purpose:**

To predict solid and micropapillary components in lung invasive adenocarcinoma using radiomic analyses based on high-spatial-resolution CT (HSR-CT).

**Materials and methods:**

For this retrospective study, 64 patients with lung invasive adenocarcinoma were enrolled. All patients were scanned by HSR-CT with 1024 matrix. A pathologist evaluated subtypes (lepidic, acinar, solid, micropapillary, or others). Total 61 radiomic features in the CT images were calculated using our modified texture analysis software, then filtered and minimized by least absolute shrinkage and selection operator (LASSO) regression to select optimal radiomic features for predicting solid and micropapillary components in lung invasive adenocarcinoma. Final data were obtained by repeating tenfold cross-validation 10 times. Two independent radiologists visually predicted solid or micropapillary components on each image of the 64 nodules with and without using the radiomics results. The quantitative values were analyzed with logistic regression models. The receiver operating characteristic curves were generated to predict of solid and micropapillary components. *P* values < 0.05 were considered significant.

**Results:**

Two features (Coefficient Variation and Entropy) were independent indicators associated with solid and micropapillary components (odds ratio, 30.5 and 11.4; 95% confidence interval, 5.1–180.5 and 1.9–66.6; and *P* = 0.0002 and 0.0071, respectively). The area under the curve for predicting solid and micropapillary components was 0.902 (95% confidence interval, 0.802 to 0.962). The radiomics results significantly improved the accuracy and specificity of the prediction of the two radiologists.

**Conclusion:**

Two texture features (Coefficient Variation and Entropy) were significant indicators to predict solid and micropapillary components in lung invasive adenocarcinoma.

**Supplementary Information:**

The online version contains supplementary material available at 10.1007/s11604-024-01534-2.

## Introduction

Lung cancer is the leading cause of cancer death worldwide [1]. Nearly 70–90% of all surgically resected lung cancers are invasive adenocarcinomas. The morphologic and pathologic manifestations of invasive adenocarcinoma (IVA) have been well characterized according to a multidisciplinary classification proposed by the International Association for the Study of Lung Cancer, the American Thoracic Society, and the European Respiratory Society (IASLC/ATS/ERS) [2]. IVA is divided into five subtypes: lepidic, acinar, papillary, solid, and micropapillary patterns. Most IVAs appear as a sequential tissue transition between two or more histologic patterns [2]. Micropapillary and solid pattern predominant tumors are known to have worse survival prognosis and higher recurrence rates. Furthermore, they are considered to have a worse prognosis even when their pattern is not predominant [3–5]. In particular, micropapillary pattern is associated with an increased risk of local recurrence in patients treated with limited resection [6]. Therefore, it is important to predict micropapillary and solid components when deciding on the surgical approach.

Preoperative diagnostic methods include percutaneous needle biopsy or bronchoscopy, but these are invasive, especially in patients with underlying diseases such as emphysema, and are sometimes difficult in patients with poor performance status. There remain problems with the accuracy and reliability of biopsy, as only a portion of the tissue is sampled. CT is a relatively simple and minimally invasive examination at the earliest stage for treating lung cancer than biopsy. Some samples do not provide a complete picture of the tumor, and the nature of the tumor may differ depending on where the sample is taken.

Imaging, on the other hand, is superior in that it provides an entire image of the tumor.

Some studies have investigated the correlation between CT morphologic and histologic features [7]. High-spatial-resolution (HSR) CT scanners, which have a spatial resolution of up to 150 mm, have been available in the clinical setting since 2017.

Recently, HSR-CT has been reported to improve diagnostic performance by subjective assessment of pathological invasiveness in adenocarcinoma due to its improved spatial resolution [8]. However, it is still difficult to distinguish subtypes of lung adenocarcinoma by morphological features. Radiomics features extracted from image data have been used in several recent studies [9].

The radiomics approach has also been used to distinguish subtypes of lung adenocarcinoma [10]. We hypothesized that the radiomics approach with improved spatial resolution of HSR-CT data could predict solid and micropapillary components in lung invasive adenocarcinoma.

The purpose of our study is to predict solid and micropapillary components in lung invasive adenocarcinoma using HSR-CT-based radiomic analysis.

## Materials and methods

### Study participants

This retrospective study was approved by our institution's internal ethics review board. Informed consent was waived for review of patient records and images. We reviewed patients who underwent surgery at a single institution between January 2018 and December 2019, and found 248 patients who underwent preoperative CT. Of these, a total of 157 patients with 159 nodules were included.

**Fig.1:**
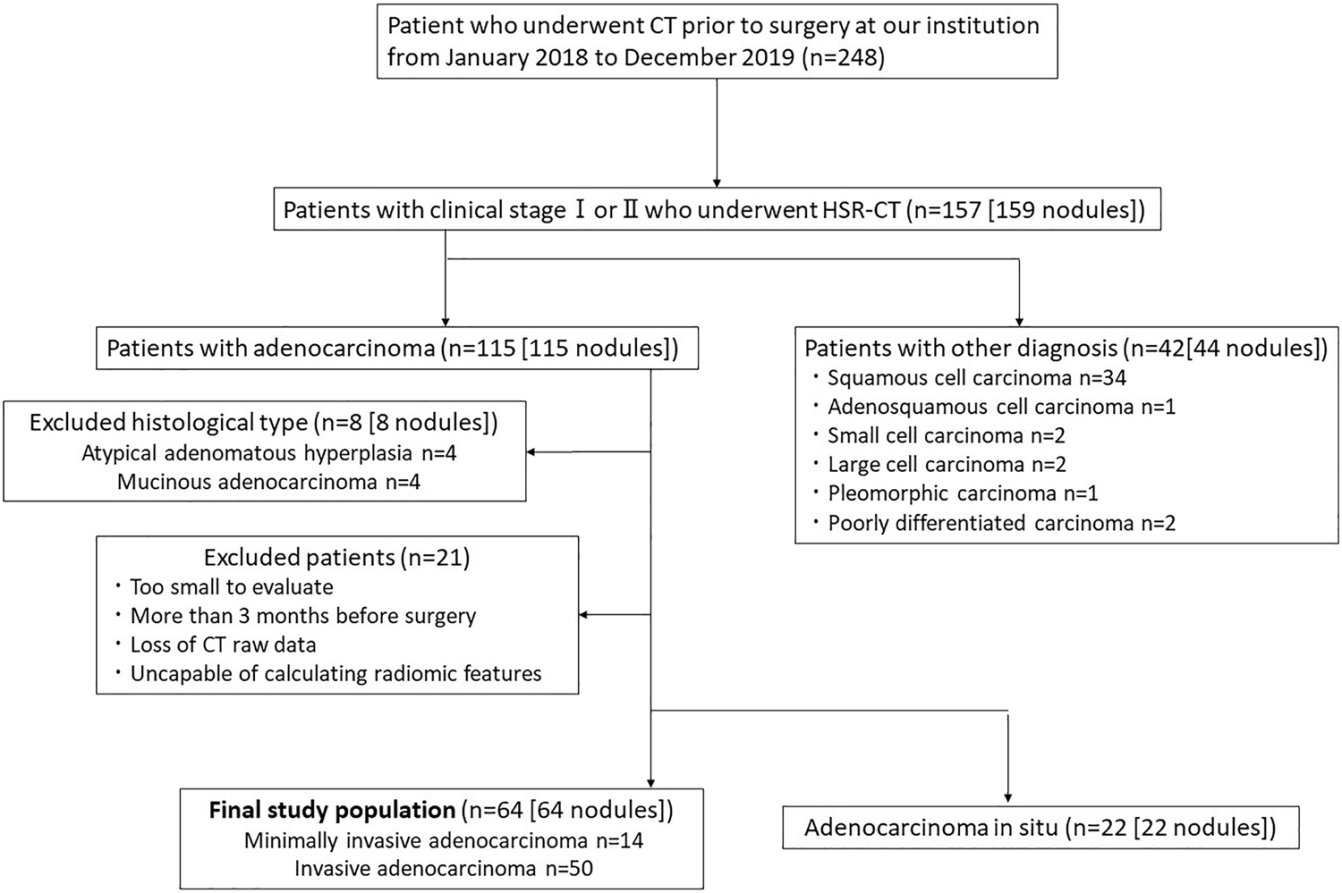
Flowchart of patient selection

The inclusion criteria were as follows (Fig. 1): (I) histologically diagnosed adenocarcinoma, (II) CT was performed with a 1024 matrix and 0.25-mm thickness, (III) clinical stage I or II lung cancer, (IV) no previous treatment, (V) age 20 years or older.

Of the 159 nodules, 115 were histologically diagnosed with adenocarcinoma. Of the 115 nodules, four with atypical adenomatous hyperplasia and four with mucinous adenocarcinoma were excluded because they differed from non-mucinous adenocarcinoma in genetic factors and cancer genesis. 21 patients (21 nodules) were excluded: patients with nodules too small to evaluate, loss of raw CT data and inability to calculate radiomic features, and those who underwent CT more than 3 months before surgery.

A total of 64 nodules with minimally invasive adenocarcinoma or invasive adenocarcinoma were finally included in our study.

### Image acquisition

All CT images were acquired on an HSR-CT scanner (Aquilion Precision: Canon Medical Systems) with 160 detector rows and 1792 detector channels. The CT parameters were as follows: tube voltage, 120 kVp; tube current, auto-exposure control; focus size, 0.6 × 0.6 mm; gantry rotation time, 0.5 s in spiral mode. Clinical images were acquired during breath-hold with full inspiration. All CT images were reconstructed with the following settings: matrix size, 1024 × 1024; slice thickness, 0.25 mm; slice interval, 0.25 mm; field of view, 34.5 cm; FC 51 (a lung algorithm) with adaptive iterative dose reduction. Volume CT dose index was 13.65 mGy ± 2.44.

### Histopathologic data

All pathologic specimens were stained with hematoxylin–eosin and evaluated by pathologists at our institution according to the multidisciplinary adenocarcinoma criteria [2]. The extent of all five growth patterns (lepidic, acinar, papillary, micropapillary, and solid) was recorded by the percentage.

### Image analysis

Commercially available software (WatchinGGO; LISIT, Co., Ltd., Tokyo, Japan) was used to segment pulmonary nodules. This software was modified for texture analysis. It can calculate radiomics features of selected two-dimensional (2D) CT images with a 1024 matrix. A total of 61 radiomic features were calculated in the segmented CT images using the software. The 61 radiomics features are listed in Online Resource 1.

**Fig.2:**
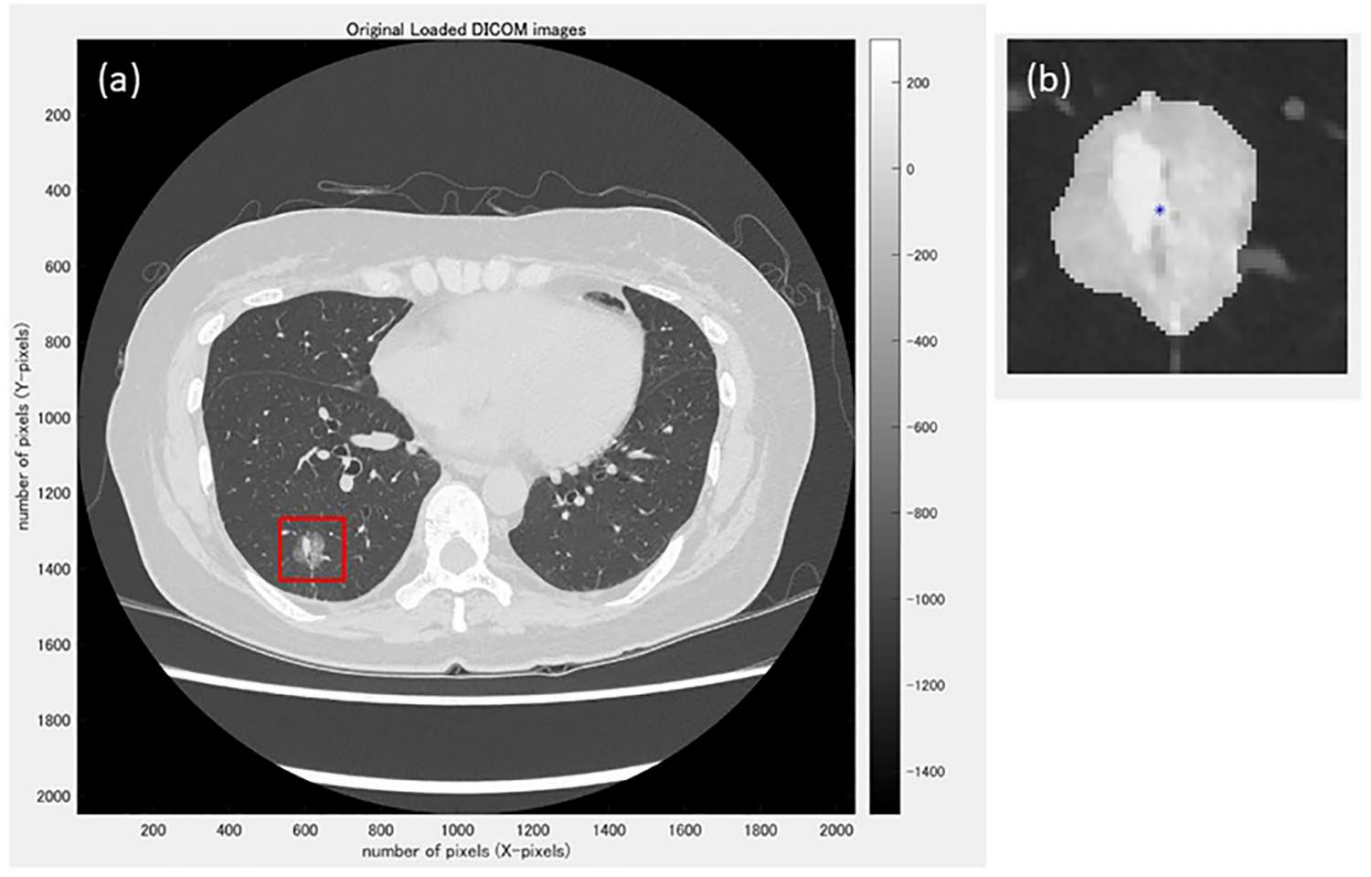
**a** A pulmonary nodule is manually surrounded by a rectangle. **b** The nodule is automatically segmented, and the ROI is manually corrected by the two radiologists as necessary. The radiomic features within the ROI are automatically calculated

Semi-automatic segmentation was performed in the maximum cross-sectional image of each tumor. The maximum cross-sectional image of each tumor was selected by a chest radiologist (MY). In the case of part-solid GGN lesions, the cross-sectional image with the maximum size of the solid component was selected. For each tumor, two independent radiologists (MT and YS) delineated regions of interest on the axial images (Fig. 2). Then, 61 radiomics features were calculated for each segmentation using our software. The average value of the radiomics features by two independent radiologists was statistically analyzed.

### Subjective image analysis

Two image review sessions were performed. In the first session, two chest radiologists (R1 (NK) and R2 (YY)) independently classified the 64 nodules into two groups (score = 1: nodules with solid and/or micropapillary components, and score = 0: those without solid or micropapillary components). They were given the following reference information beforehand; micropapillary and solid patterns are more likely to be: (a) larger (total size≧2.5 cm), and/or (b) solid component predominant, and/or (c) spiculation or lobulation, whereas lepidic pattern is likely to contain a greater proportion of ground-glass opacities and/or an air bronchogram [7]. In the second session, performed 1 month after the first session, the same 64 nodules were reclassified into the two above-mentioned groups on the basis of the radiomics results which were significant indicators to predict the score = 1. At this time, they were also presented with the AUC value.

### Statistical analysis

Statistical analyses were performed using commercially available software (MedCalc® Statistical Software version 20.216, Ostend, Belgium). Python 3.8.6 and scikit-learn 0.24.1 were used for the radiomics analysis.

The 61 radiomic features were filtered by least absolute shrinkage and selection operator (LASSO) regression with tenfold cross-validation 10 times. We determined feature importance by counting the number of times the feature had non-zero regression coefficients through repeated cross-validation. We created the radiomics features using a linear combination of the selected features. A radiomics score was calculated for each patient using a linear combination of the selected features, weighted according to their coefficients. For each higher relevant feature, the cutoff value that yielded the largest difference in the number of patients with and without solid and micropapillary components was determined using the receiver operating characteristic (ROC) method. Optimal cutoff values were determined for each variable separately using the Youden index (the maximum value of sensitivity and specificity). Associations between solid and micropapillary components and each binary group (score = 0 or score = 1) designated by the cutoff value for the ten radiomics features were evaluated by univariate logistic regression analysis. Significant features identified by univariate analysis were included in multiple logistic regression (stepwise method; *P* value of 0.05 or less was used for entry into the model and *P* value greater than 0.1 was selected for removal).

Diagnostic performance for predicting solid and micropapillary components was analyzed using ROC curves: sensitivity, specificity, and area under the curve (AUC). We used the “Comparison of ROC curves” of the MedCalc software to test the difference in the AUCs between predictive performance with and without radiomics features. Comparisons of accuracy, sensitivity, and specificity were performed using McNemar’s test. *P* value < 0.05 was considered significant.

## Results

### Clinical and pathological characteristics

Table1 shows the clinical and pathologic characteristics. Of the 64 patients, 16 patients had solid or micropapillary components and 48 patients had no solid or micropapillary components.

**Table 1:** Clinical and pathological characteristics

Characteristics		
Sex		
	Male	38 (59.4%)
	Female	26 (40.6%)
Age		70.0 (42–89)
Histologic subtype		
Solid( +)/micropapillary( +)		1
Solid( +)/micropapillary(-)		14
Solid(-)/micropapillary( +)		1
Solid(-)/micropapillary(−)		48
Radiologic appearance		
Pure GGN^a^		3 (4.7%)
Part solid GGN^a^		44 (68.7%)
Solid nodule		17 (26.6%)

^a^GGN: ground-glass nodule

### Prediction of solid and micropapillary components using radiomic analysis

**Fig.3:**
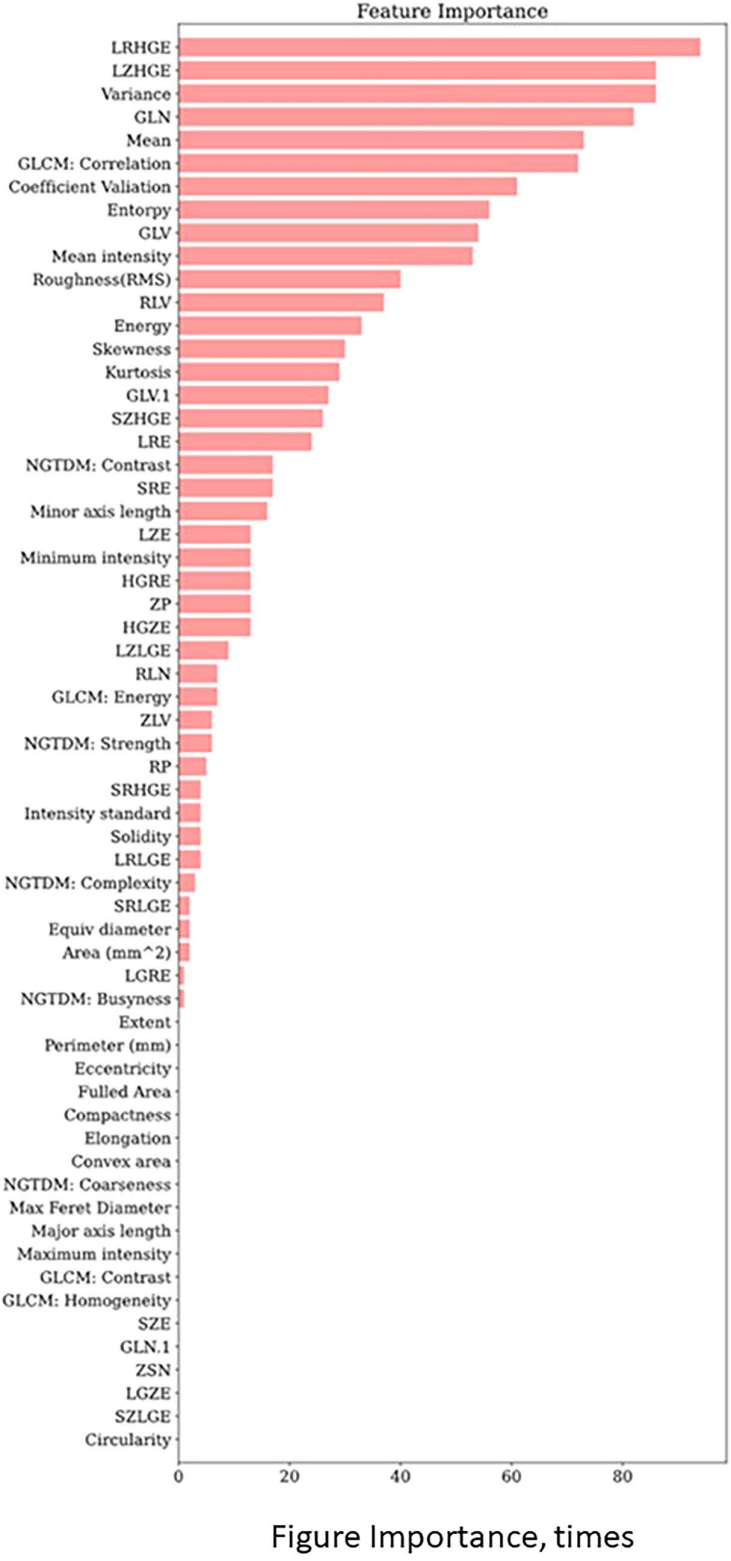
Importance of radiomic features for predicting solid and micropapillary components. We defined a radiomic feature as important if it had a non-zero LASSO regression coefficient over the repeated cross-validation. The maximum number of repetitions was 100. *LRHGE* large run high gray-level emphasis, *LZHGE* large zone high gray-level emphasis, *GLN* gray level non-uniformity, *GLCM* gray level co-occurrence matrix, *GLV* gray level variance, *RLV* run length variance, *SZHGE* small zone low gray-level emphasis, *LRE* long run emphasis, *NGTDM* neighborhood gray-tone-difference matrix, *SRE* short run emphasis, *LZE* large zone emphasis, HGRE high gray-level run emphasis, *ZP* zone percentage, *HGZE* high gray-level zone emphasis, LZLGE large zone low gray-level emphasis, *RLN* run-length non-uniformity, *ZLV* zone level variance, *RP* run percentage, *SRHGE* short run high gray-level emphasis, *LRLGE* long run low gray-level emphasis, *SRLGE* short run low gray-level emphasis, *LGRE* low gray-level run emphasis, *SZE* small zone emphasis, *ZSN* zone size non-uniformity, *LGZE* low gray-level zone emphasis, *SZLGE* small zone low gray-level emphasis, *GLCM* gray-level co-occurrence matrix, *GLN* gray-level non-uniformity, *LRHGE* large run high gray-level emphasis, *GLV* gray level variance, *LZHGE* large zone high gray-level emphasis

We extracted ten optimal radiomic features for predicting solid and micropapillary components: LRHGE, LZHGE, Variance, GLN, Mean, GLCM: Correlation, Coefficient Variation, Entropy, GLV and Mean intensity (Fig. 3). The formula used to calculate the ten radiomic features is shown in Online Resource 2.

We determined cutoff values for each of the ten features using ROC analysis (Table 2). Univariable logistic regression analyses of these features revealed that all ten radiomic features were statistically significant for predicting micropapillary and solid patterns. Multiple logistic regression analysis revealed that two features (Coefficient of Variation and Entropy) were independent indicators associated with solid and micropapillary components (odds ratio, 30.5 and 11.4; 95% confidence interval, 5.1–180.5 and 1.9–66.6; and *P* = 0.0002 and 0.0071, respectively). The area under the curve for predicting solid and micropapillary components was 0.902 (95% confidence interval, 0.802 to 0.962).

**Table 2:** Cutoff values of radiomic features for predicting solid and micropapillary components in lung invasive adenocarcinoma

Features	Cut-off value	Mean ± SD (standard deviation)
Mean intensity		
Score = 0 (*n* = 40)		− 498.5 ± 98.9
Score = 1 (*n* = 24)	> − 3.46 × 10^2^	− 206.2 ± 140.8
Coefficient variation		
Score = 0 (*n* = 44)		− 0.51 ± 0.64
Score = 1 (*n* = 20)	≦− 1.13	− 1.73 ± 0.50
Mean		
Score = 0 (*n* = 42)		16.3 ± 2.3
Score = 1 (*n* = 22)	> 19.7	23.4 ± 2.6
Variance		
Score = 0 (*n* = 42)		1.21 × 10^2^ ± 31.7
Score = 1 (*n* = 22)	> 1.81 × 10^2^	2.46 × 10^2^ ± 60.3
Entropy		
Score = 0 (*n* = 40)		3.12 ± 0.14
Score = 1 (*n* = 24)	> 3.32	3.5 ± 0.08
GLCM^a^: Correlation		
Score = 0 (*n* = 32)		0.94 ± 2.2 × 10^−2^
Score = 1 (*n* = 32)	> 0.96	0.97 ± 5.3 × 10^−3^
GLN^b^		
Score = 0 (*n* = 40)		5.1 × 10^–2^ ± 6.4 × 10^−3^
Score = 1 (*n* = 24)	> 6.1 × 10^−2^	7.8 × 10^–2^ ± 1.4 × 10^−2^
LRHGE^c^		
Score = 0 (*n* = 40)		6.26 × 10^2^ ± × 1.92 × 10^2^
Score = 1 (*n* = 24)	> 9.80 × 10^2^	1.39 × 10^3^ ± 2.63 × 10^2^
GLV^d^		
Score = 0 (*n* = 41)		1.7 × 10^–2^ ± 4.6 × 10^−3^
Score = 1 (*n* = 23)	> 2.5 × 10^−2^	5.5 × 10^–2^ ± 2.4 × 10^−2^
LZHGE^e^		
Score = 0 (*n* = 39)		1.36 × 10^4^ ± 1.05 × 10^4^
Score = 1 (*n* = 25)	> 3.53 × 10^4^	1.82 × 10^5^ ± 1.58 × 10^5^

^a^Gray-level co-occurrence matrix

^b^gray level non-uniformity

^c^long run high gray-level emphasis

^d^Gray level variance

^e^large zone high gray-level emphasis

### Prediction of solid and micropapillary component by radiologists with and without radiomics results

The prediction by the two chest radiologists without the radiomic results were as follows: accuracy, sensitivity, specificity, positive predictive value (PPV), negative predictive value (NPV): R1 and R2, 54.7 and 54.7%, 100 and 93.7%, 39.6 and 41.7%, 35.6 and 34.9%, 100 and 95.2%, respectively). The radiomics results improved the accuracy and specificity significantly, while it worsened the sensitivity of R2, not significantly (Table 3). The AUC values for prediction with radiomics results were higher than without radiomics, but not significant: 0.698 (95% CI, 0.580–0.806) vs. 0.802 (0.684–0.891), *P* = 0.14 and 0.677 (0.549–0.789) vs. 0.823 (0.707–0.907), *P* = 0.07.

**Table 3:** Accuracy, sensitivity, specificity of the radiologists with and without the radiomics results

		Radiomics results		Difference	95%CI^a^	*P* value
		(−)	( +)			
Accuracy	R1	54.7%	70.3%	+ 15.6%	+ 6.7− + 24.5	0.0002
	R2	54.7%	85.9%	+ 31.2%	+ 17.6− + 44.9	0.0001
Sensitivity	R1	100%	100%	± 0		1
	R2	93.7%	75%	− 18.7%	− 37.9− + 0.38	0.25
Specificity	R1	39.6%	60.4%	+ 20.8%	+ 9.3- + 32.3	0.0002
	R2	41.7%	89.6%	+ 47.9%	+ 33.8− + 62.1	< 0.0001

^a^Confidence interval

## Discussion

Our study showed that radiomic analysis of HSR-CT data with 1024 matrix could achieve relatively accurate prediction performance of micropapillary and solid patterns. (AUC value of 0.90; 95% CI: 0.80–0.96). Considering that micropapillary and solid pattern predominant tumors had worse survival prognosis and higher recurrence rates, the good performance of our radiomics model could be an effective tool for prognosis prediction and clinical treatment planning decision, especially in cases where surgery or biopsy is not feasible. In particular, it may be more useful when a tumor has only a small portion of the solid and micropapillary pattern, which are difficult to detect by biopsy. Tissue biopsy is essential to examine the histopathology of a tumor. However, the spatially and temporally heterogeneous properties of tumors remain a problem for the accuracy and reliability of biopsy using only a portion of the tissue. If the CT-based radiomics approach, which is a simple and non-invasive examination, can predict the pathological diagnosis, the clinical benefit to patients is also considered to be significant.

Some previous studies have attempted to predict subtypes of lung adenocarcinoma. Some studies have reported radiologic features of each subtype, but they are not specific [7]. Recently, a radiomic approach has been used to predict micropapillary and solid patterns. Bingxi demonstrated that the radiomic method achieved good prediction performance of micropapillary and solid patterns (AUC value of 0.75; 95% CI: 0.65–0.85) [11]. Yunyu X et al. [12] reported that the combination of radiomics and clinical features had a statistically better prediction performance of micropapillary pattern (AUC = 0.739; 95% CI: 0.594–0.885) than radiomics analysis alone (AUC = 0.722; 95% CI: 0.574–0.870). A previous study showed that the radiomic approach achieved a higher PPV than radiologists for solid or micropapillary-predominant adenocarcinoma [13]. We performed radiomics analysis using HSR-CT data and achieved better predictive performance than conventional CT data. HSR-CT can provide higher image quality than conventional CT by improving spatial resolution (0.15 mm in plane, 0.20 mm through plane) and reducing artifacts due to undershoot [14]. A large matrix size maintained spatial resolution and improved image quality and assessment of lung disease [15]. The 1024 matrix size can provide detailed texture features compared to conventional CT. Tao et al. reported that the radiomics features with HRCT data of small pixel size achieved higher AUC in predicting the invasiveness of GGNs than larger pixel size [16]. The accuracy of radiomics features with HSR-CT data may improve the accuracy of predicting solid and micropapillary components.

**Fig.4:**
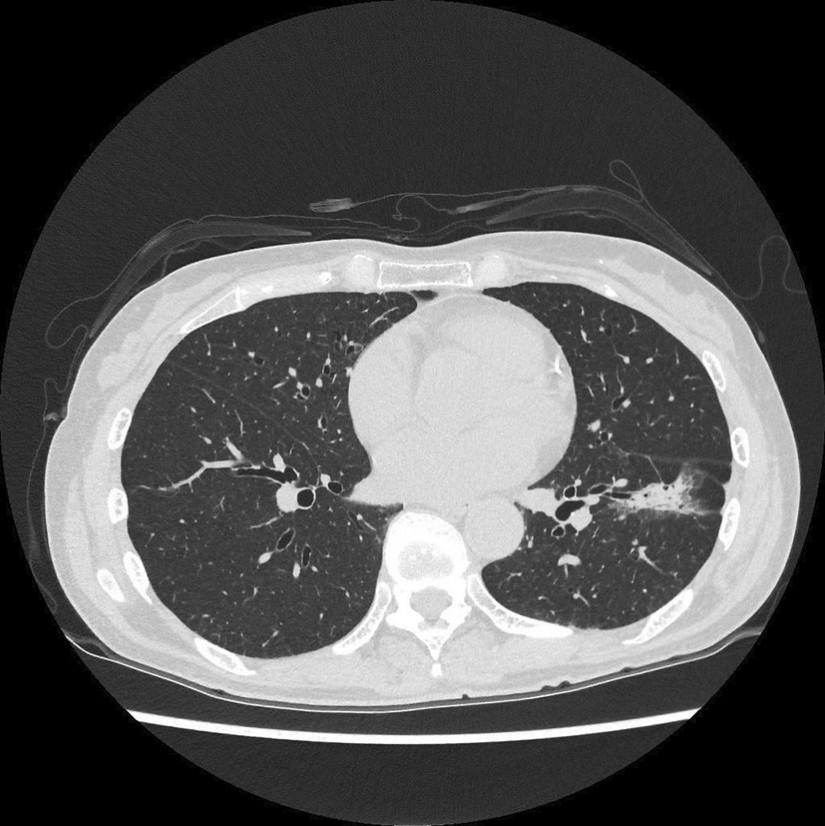
There is a part-solid nodule in the left lower lobe. It is a lesion with a large proportion of solid component and it seems to be suggestive of a solid or micropapillary component. However, it has some degree of GGO and it is difficult to predict visually with certainty. Pathologically, it was a nodule without solid or micropapillary component. The radiologists predicted that it had a solid or micropapillary component. By referring to the radiomics results, they were able to predict correctly. The radiomics result entropy 3.36, coefficient of variance: − 0.817

The radiomics results significantly improved the specificity and, not significantly, the readers' AUC value. Visual assessment seems to have a tendency to predict that nodules have micropapillary or solid component, because many lesions had findings suggestive of micropapillary or solid components, such as solid portion or spiculation. However, nodules consisting mainly of solid components can also be those without micropapillary or solid portions. Therefore, there are many false positives in visual assessment, and radiomics results can significantly reduce them (Fig. 4).

**Fig.5:**
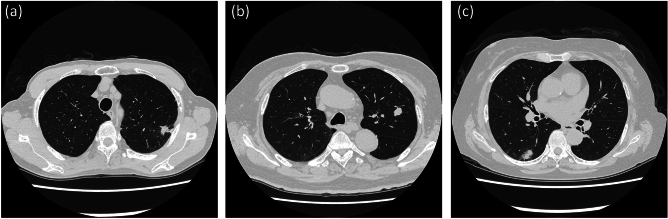
The three nodules with micropapillary and solid component: The radiomics method of this study predicted that they did not have micropapillary and solid component. The radiologists correctly predicted them without the radiomics results. After referring to the radiomics results, one did not change her prediction: it was correct, and the other did change it: it was incorrect. Thus, the decision to use radiomics results sometimes differs between radiologists. **a** A part-solid nodule of the left upper lobe with a small percentage of ground-glass opacity and spiculation. (The radiomics results: entropy 3.25, coefficient of variance − 1.34). **b** A solid nodule of the left upper lobe with almost no spiculation. (The radiomics results: entropy 3.19, coefficient of variance − 1.55). **c** A part-solid nodule of the right lower lobe, composed mainly of solid components and with spiculation. (The radiomics results: entropy 3.36, coefficient of variance − 0.877)

**Fig.6:**
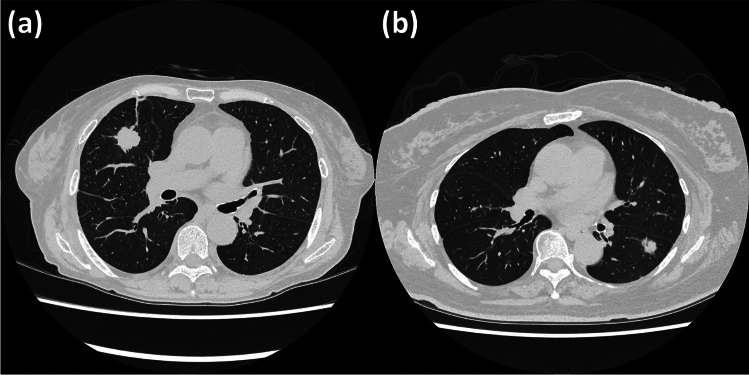
**a** There is a solid nodule in the right middle lobe. It has solid component. (The radiomics results: entropy 3.43, coefficient of variance − 1.93) The radiologists correctly predicted with and without the radiomics results. **b**There is a solid nodule in the left lower lobe. It is a nodule without solid or micropapillary component. (The radiomics results: entropy 3.13, coefficient of variance − 0.918) The radiologists predicted wrongly without the radiomics results. After referring to the radiomics results, one did not change her prediction: it was incorrect, and the other did change it: it was correct. The two nodules are both visually solid and there appears to be little difference in appearance between them. However, **a** is a nodule with solid components, whereas **b** is without solid or micropapillary components. Although the radiomics method predicted correctly, it may be difficult to decide whether to accept the results because both are visually suspected to be nodules with solid or micropapillary components

On the other hand, it may be a problem that the criteria for whether to use the radiomics results vary from one radiologist to another (Fig. 5). For example, it may be difficult to use the radiomics results if they do not agree with the visual assessment, even though the performance of the radiomics method is good (Fig. 6). That’s because the basis for radiomics predictions is unclear. It is likely to be difficult to prove, and it may be necessary to set standards for whether to use radiomics results.

Lung adenocarcinomas are usually heterogeneous with multiple subtypes. Thus, extracted radiomics values do not necessarily reflect the characteristics of each subtype itself, but those of the entire lesion with multiple subtypes. To reduce the influence of mixed subtypes, Li-Wei C et al. performed radiomics analysis with “near-pure” histopathological subtypes (a predominant subtype > 70% of tumor volume) and achieved high sensitivity and moderate specificity [17].

In this study, higher entropy and coefficient of variation were significant indicators to predict solid and micropapillary components in lung invasive adenocarcinoma. According to some previous studies, higher histogram entropy was associated with worse outcome. This may reflect that micropapillary and solid components have poor prognosis [18, 19]. Entropy and coefficient of variation are first-order statistics that describe the distribution of voxel intensities within the image region. Entropy describes the uncertainty or randomness of the image values. The coefficient of variation is the ratio of the standard deviation to the mean, and the higher the coefficient of variation, the greater the dispersion. This indicates that tumors with solid and micropapillary components have heterogeneity, and even invisible heterogeneity can be identified with the radiomics approach. In this study, detailed radiomics was performed using HSR-CT data with 1024 matrix, but the results were based on 2D analysis. The pathological tumor heterogeneity is complex, and further analysis is needed to develop dedicated software for three-dimensional (3D) texture analysis on HSR-CT with 1024 matrix, and to investigate the relationship between micropapillary and solid component and 3D texture analysis using a large cohort in the future.

Our study has several limitations. First, this study was based on 2D texture as described above. 3D texture analysis is desirable to analyze the complex heterogeneity of tumors. However, we analyzed not a whole lesion of the tumors but cross-sectional CT images where the solid portion is the maximum because currently there is no 3D texture analysis software for data on HSR-CT with large matrix size. Second, this study was a retrospective study at a single institution, and the number of cases with micropapillary was small. Because of the small data in this study, ten-fold cross-validation was performed for the evaluations. Further studies with larger cohorts including micropapillary and solid patterns are needed to confirm our results. Third, we used only a single texture analysis software. Texture features are known to be significantly affected by the CT acquisition protocol and pre-processing methods [20]. The way of segmentation and selection of cross-sections may vary the radiomic features. In this study, there was no influence of the selection of the cross-sections, because the two radiologists segmented the same cross-sectional CT images that were selected in advance.

In conclusion, two texture features (coefficient variation and entropy) from HSR-CT with 1024 matrix were significant indicators to predict solid and micropapillary components in lung invasive adenocarcinoma. This may be useful to predict the prognosis of each patient and determine a more appropriate treatment plan. However, since different radiologists have different criteria for using radiomics results, it may be necessary to build an explanatory radiomics prediction model and establish criteria for our future use of radiomics results.

### Supplementary Information

Below is the link to the electronic supplementary material.

Supplementary file1 (DOCX 16 KB)

Supplementary file2 (DOCX 20 KB)
