# Supplementary material for: Prediction of solid and micropapillary components in lung invasive adenocarcinoma: radiomics analysis from high-spatial-resolution CT data with 1024 matrix
Source: Jpn J Radiol. 2024 Feb 28;42(6):590–8. doi: 10.1007/s11604-024-01534-2 (PMC11139717; doi:10.1007/s11604-024-01534-2)
Supplement: Supplementary file 1 — Supplementary file1 (DOCX 16 KB) [file 11604_2024_1534_MOESM1_ESM.docx]

| **Shape feature**  Circularity  Roughness  Elongation  Compactness  Eccentricity  Solidity  Extent  **Size Feature**  Area  Fulled Area  Convex Area  Perimeter  Max Feret Diameter  Equiv diameter  Major axis length  Minor axis length  **Intensity Feature**  Minimun  Maximum  Mean intensity  Intensity standard  Coefficient valiation  **Histogram Features**  Mean  Variation  Skewness  Kurtosis  Energy  Entropy | **GLCM & NGTDM**  GLCM: Contrast  GLCM: Correlation  GLCM: Energy  GLCM: Homogeneity  NGTDM: Coarseness  NGTDM: Contrast  NGTDM: Busyness  NGTDM: Complexity  NGTDM: Strength  **GLRLM**  SRE  LRE  GLN  RLN  RP  LGRE  HGRE  SRLGE  SRHGE  LRLGE  LRHGE  GLV  RLV  **GLSZM**  SZE  LZE  GLN  ZSN  ZP  LGZE  HGZE  SZLGE  SZHGE  LZLGE  LZHGE  GLV  ZLV |
| --- | --- |

GLCM: gray-level co-occurrence matrix, NGTDM: neighborhood gray-tone-difference matrix, GLRLM: grey-level run length matrix, SRE: short run emphasis, LRE: long run emphasis, GLN: gray level non-uniformity, RLN: run-length nonuniformity, RP: run percentage, LGRE: low gray-level run emphasis, HGRE: high gray-level run emphasis, SRLGE: short run low gray-level emphasis, SRHGE: short run high gray-level emphasis, LRLGE: long run low gray-level emphasis, LRHGE: long run high gray-level emphasis, GLV: gray level variance, RLV: run length variance, GLSZM: gray-level size zone matrix, SZE: small zone emphasis, LZE: large zone emphasis, GLN: gray level non-uniformity, ZSN: zone size non-uniformity, ZP: zone percentage, LGZE: low gray-level zone emphasis, HGZE: high gray-level zone emphasis, SZLGE: small zone low gray-level emphasis, SZHGE: small zone low gray-level emphasis, LZLGE: large zone low gray-level emphasis, LZHGE: large zone high gray-level emphasis, GLV: gray level variance, ZLV: zone level variance
