# Supplementary material for: Prediction of solid and micropapillary components in lung invasive adenocarcinoma: radiomics analysis from high-spatial-resolution CT data with 1024 matrix
Source: Jpn J Radiol. 2024 Feb 28;42(6):590–8. doi: 10.1007/s11604-024-01534-2 (PMC11139717; doi:10.1007/s11604-024-01534-2)
Supplement: Supplementary file 2 — Supplementary file2 (DOCX 20 KB) [file 11604_2024_1534_MOESM2_ESM.docx]

**Radiomic Features**

**LRHGE: Long Run High Gray Level Emphasis**

Notations

- $\mathbf{P}(i,j\mid\theta)$ is the run length matrix of direction $\theta$
- $p(i,j\mid\theta)$ is the normalized run length matrix
- $N_{g}$ is the number of discrete intensity values in the image
- $N_{r}$ is the number of discrete run lengths in the image
- $N_{p}$ is the number of voxels in the image

***LRHGLRE***=$\frac{\sum_{i=1}^{N_{g}} \sum_{j=1}^{N_{r}} \mathbf{P}(i,j\mid\theta)i^{2}j^{2}}{\sum_{i=1}^{N_{g}} \sum_{j=1}^{N_{r}} \mathbf{P}(i,j\mid\theta)}$

LRHGLRE (LRHGE) measures the joint distribution of long run lengths with higher gray-level values.

**LZHGE: Large Zone High Gray Level Emphasis**

***F_szm.lzhge_***=$\frac{1}{N_{s}}\sum_{i=1}^{N_{g}} \sum_{j=1}^{N_{z}} i^{2}j^{2}s_{ij}$

- Ng is the number of discretized gray levels existing in the ROI signal intensity mask
- Nz is the maximum zone size of the voxel group.
- Ns is the total number of zones.

This feature evaluates the distribution of zone numbers for gray levels. If the zones are evenly distributed along the gray levels, the feature value will be low.

**Variance**

***variance***=$\frac{1}{N}\sum_{i=1}^{N} (\mathbf{X}(i)-\hat{X})^{2}$

Variance is the mean of the squared distances of each intensity value from the mean value. This is a measure of the spread of the distribution about the mean.

**GLN: Gray Level Non-Uniformity**

***GLN***=$\frac{\sum_{i=1}^{N_{g}} \left( \sum_{j=1}^{N_{r}} \mathbf{P}(i,j\mid\theta) \right)^{2}}{\sum_{i=1}^{N_{g}} \sum_{j=1}^{N_{r}} \mathbf{P}(i,j\mid\theta)}$

GLN measures the similarity of gray-level intensity values in the image, where a lower GLN value correlates with a greater similarity in intensity values.

**Mean Intensity**

***mean***=$\frac{1}{N}\sum_{i=1}^{N} \mathbf{X}(i)$

The average gray level intensity within the ROI.

**GLCM: Gray-Level Co-occurrence Matrix Correlation**

***correlation***=$\frac{\sum_{i=1}^{N_{g}} \sum_{j=1}^{N_{g}} p(i,j)ij-\mu_{x}(i)\mu_{y}(j)}{\sigma_{x}(i)\sigma_{y}(j)}$

Notations:

- $\mathbf{P}(i,j)$ is the co-occurence matrix for $\delta$ (distance) and $\alpha$ (angle)
- $p(i,j)$ is the normalized co-occurrence matrix.
- $N_{g}$ is the number of discrete intensity levels in the image
- $p_{x}(i)=\sum_{j=1}^{N_{g}} P(i,j)$ is the marginal row probabilities.
- $p_{y}(j)=\sum_{i=1}^{N_{g}} P(i,j)$ is the marginal column probabilities.
- $\mu_{x}=\sum_{i=1}^{N_{g}} \sum_{j=1}^{N_{g}} P(i,j)i$ is the mean gray level intensity of $p_{x}$
- $\mu_{y}=\sum_{i=1}^{N_{g}} \sum_{j=1}^{N_{g}} P(i,j)j$ is the mean gray level intensity of $p_{y}$
- $\sigma_{x}$ is the standard deviation of $p_{z}$
- $\sigma_{y}$ is the standard deviation of $p_{y}$
- $p_{x+y}(k)=\sum_{i=1}^{N_{g}} \sum_{j=1}^{N_{g}} P(i,j)$, where $i+j=k$
- $p_{x-y}(k)=\sum_{i=1}^{N_{g}} \sum_{j=1}^{N_{g}} P(i,j)$, where $|i-j|=k$
- $HX=-\sum_{i=1}^{N_{g}} p_{x}(i)\log_{2}\left( p_{x}(i)+\epsilon\right)$ is the entropy of $p_{x}$
- $HY=-\sum_{j=1}^{N_{g}} p_{y}(j)\log_{2}\left( p_{y}(j)+\epsilon\right)$ is the entropy of $p_{y}$
- $HXY=-\sum_{i=1}^{N_{g}} \sum_{j=1}^{N_{g}} p(i,j)\log_{2}(p(i,j)+\epsilon)$ is the entropy of $p(i,j)$
- $HXY1=-\sum_{i=1}^{N_{g}} \sum_{j=1}^{N_{g}} p(i,j)\log_{2}\left( p_{x}(i)p_{y}(j)+\epsilon\right)$
- $HXY2=-\sum_{i=1}^{N_{g}} \sum_{j=1}^{N_{g}} p_{x}(i)p_{y}(j)\log_{2}\left( p_{x}(i)p_{y}(j)+\epsilon\right)$

Correlation is a value between 0 (uncorrelated) and 1 (perfectly correlated)

showing the linear dependency of gray level values to their respective voxels in

the GLCM.

**Coefficient Variation:**

***CV***=$\frac{\text{ Standard Deviation }}{\text{ Mean }}$

Coefficient Variation (CV) is a dimensionless measure of data variability relative to the mean. It's calculated as the ratio of the standard deviation to the mean. A higher CV indicates greater data variability, while a lower CV suggests less variability. In image analysis, it helps assess the diversity of image features.

**Entropy**

***entropy***=$-\sum_{i=1}^{N_{g}} \sum_{j=1}^{N_{g}} p(i,j)\log_{2}(p(i,j)+\epsilon)$

Entropy is a measure of the randomness/variability in neighborhood intensity values.

**GLV: GrayLevelVariance**

***GLV***=$\sum_{i=1}^{N_{g}} \sum_{j=1}^{N_{r}} p(i,j\mid\theta)(i-\mu)^{2}$

$$\mu=\sum_{i=1}^{N_{g}} \sum_{j=1}^{N_{r}} p\left( i,j \mid\theta\right)i$$

GLV measures the variance in gray level intensity for the runs.

**Histogram Mean**

The formula for calculating the mean (average) using a 64-bin histogram analysis is as follows:

***Mean***=$\frac{\sum_{i=1}^{64} \left( x_{i}\cdot f_{i} \right)}{N}$

Where:

- $x_{i}$ represents the center value of each bin.
- $f_{i}$ represents the frequency (count) of data points in each bin.
- $N$ is the total number of data points or the sum of frequencies in the histogram.

This formula allows you to compute the mean value of the data using the bin center values and frequencies from the histogram.
